# Deploying viscosity and starch polymer properties to predict cooking and eating quality models: A novel breeding tool to predict texture

**DOI:** 10.1016/j.carbpol.2021.117766

**Published:** 2021-05-15

**Authors:** Reuben James Q. Buenafe, Vasudev Kumanduri, Nese Sreenivasulu

**Affiliations:** a Grain Quality and Nutrition Center, International Rice Research Institute, Los Baños, Laguna, 4031, Philippines; b School of Chemical, Biological, Materials Engineering and Sciences, Mapua University, Muralla St., Intramuros, Manila, 1002, Philippines; c Piatrika Biosystems, Cambridge, UK

**Keywords:** Cooking and eating quality, Random forest model, Indica, Japonica

## Abstract

Acceptance of new rice genotypes demanded by rice value chain depends on premium value of varieties that match consumer demands of regional preferences. High throughput prediction tools are not available to breeders to classify cooking and eating quality (CEQ) ideotypes and to capture texture of varieties. The pasting properties in combination with starch properties were used to develop two layered models in order to classify the rice varieties into twelve distinct CEQ ideotypes with unique sensory profiles. Classification models developed using random forest method depicted the overall accuracy of 96 %. These CEQ models were found to be robust to predict ideotypes in both Indica and Japonica diversity panels grown under dry and wet seasons and across the years. We conducted random forest modeling using 1.8 million high density SNPs and identified top 1000 SNP features which explained CEQ model classification with the accuracy of 0.81. Furthermore these CEQ models were found to be valuable to predict textural preferences of IRRI breeding lines released during 1960–2013 and mega varieties preferred in South and South East Asia.

## Highlights

• Multivariate analysis was used to develop twelve cooking and eating quality classes.
• Two-layered random forest model was used to predict rice classification.
• High classification accuracy of cooking and eating quality ideotypes were obtained.
• Mismatches from IRRI-released and consumer-preferred lines was capture by the model.

## 1 Introduction

Rice (*Oryza sativa* L.) is a staple food for more than half of the world’s population primarily preferred in Asia and its demand for food consumption is growing in Africa (Bandumula, 2018; Tilman, Balzer, Hill, & Befort, 2011). To address food security, breeders have developed several varieties with higher yield potentials but often ignoring the grain quality with the exception of few mega-varieties possessing superior grain quality attributes widely cultivated as of today (Pang et al., 2016; Zeng et al., 2017). With improvement in Asian economy and rapid raise in urbanization, consumers are more willing to pay premium for premium quality. Considering both the needs of the farmers and consumers there is a need to screen rice varieties to predict CEQ and thus demanding the breeders to consider CEQ and textural preferences as one of their breeding objectives in developing new rice varieties (Calingacion et al., 2014; Pang et al., 2016). Breeding programs traditionally capture CEQ and textural properties through proxy traits such as measuring amylose content (AC) as stand alone, or assessment of gel consistency (GC) and gelatinization temperature (GT) to distinguish degree of hardness within high amylose rice and to predict cooking time, respectively (Cuevas, Domingo, & Sreenivasulu, 2018; Custodio et al., 2019). However using AC, GC and GT as proxy traits, breeding programs are not able to capture the entirety of textural preferences within *Indica* germplasm. To solve this problem, accurate and detailed evaluation tools are needed for the selection of high quality rice (Chandra, Takeuchi, & Hasegawa, 2012), in the background of high yield potential. Global preferences of CEQ are difficult to define in rice because of diversified regional preferences of consumers. Despite numerous measures of grain quality, the best indicators of CEQ are better perceived through the importance of organoleptic attributes of cooked rice, which can be characterized via sensory evaluation (Bett-Garber et al., 2001; Champagne et al., 1999). Sensory properties of the varieties with intermediate-high AC can be clearly distinguished through sensory panel and through visco-elastic properties (Anacleto et al., 2015; Champagne et al., 2010; Cuevas et al., 2018; Pang et al., 2016). However, sensory evaluation is not as rigorously used as a tool as routine grain quality traits in phenotyping rice varieties due to lack of throughput (Anacleto et al., 2015).

Presently, rapid visco-analyzer (RVA), a high throughput analytical instrument, can be deployed to measure rice cooking quality by assessing viscosity fingerprints. RVA properties are also correlated with sensory qualities and can be used to predict different grain quality classes of rice varieties (Bett-Garber et al., 2001; Champagne et al., 1999; Pang et al., 2016; Zhu et al., 2018). RVA also captures the retrogradation features reflecting the keeping quality (Champagne et al., 1999). Although milled rice comprises more than 90 % of starch, varieties differ in its composition of amylose and amylopectin polymers (Butardo et al., 2017; Li & Gilbert, 2018) which attributes to the variation in textural properties (Misra et al., 2018) such as degree of hardness (Yang et al., 2016) and stickiness (Cameron & Wang, 2005). Furthermore, starch pasting properties were proven to be influenced by the molecular weights of amylopectin (Kowittaya & Lumdubwong, 2014). Hence, another important CEQ indicator is the starch molecular structure which can be rapidly determined through size-exclusion chromatography (SEC) (Ward, Gao, de Bruyn, Gilbert, & Fitzgerald, 2006). RVA properties have been utilized to accurately distinguish CEQ between *Indica* and *Japonica* varieties by employing multivariate techniques (Molina, Jimenez, Sreenivasulu, & Cuevas, 2019; Zhu et al., 2018). However, there have been no models developed yet utilizing the RVA fingerprints and starch molecular properties solely or in combination to predict distinct CEQ ideotypes and as well to link genome-phenome data to predict the CEQ models. These derived tools to identify consumer-preferred varieties with superior texture matching to the demand of regional preferences (Pang et al., 2016), likely to shed important insights to capture textural preferences.

This study aims to utilize RVA and starch molecular properties to develop bi-layered models to accurately predict the CEQ classification of breeding material and to identify high quality *Indica* rice varieties matching sensory characteristics of texture preferred in the target geographic regions by consumers. In addition, high-density genotyping data available from *Indica* germplasm were used to identify top feature SNPs through modeling to predict the classifiers.

## 2 Methods

### 2.1 Rice varieties

A (*n* = 301) set of rice accessions (*Indica* Diversity Panel1) was selected covering wide geographic distribution and high genetic diversity. These accessions were planted and grown under field conditions at IRRI during the dry season of 2014 by following the standard agronomic practices. The paddy grains were harvested at maturity and equilibrated to 14 % moisture content. The grains were subjected to dehulling (Rice sheller THU-35A, satake Corporation, Hiroshima, Japan) and milling (Grainman 60-230-60-2AT, Grain Machinery Mfg. Corp., Miami, USA) prior to analysis. The grains were powdered (Cyclone Sample Mill 3010-039, Udy Corporation, Fort Collins, USA) for different biochemical analyses.

Along with this, two (*n =* 316, *n* = 318) sets of *Indica* rice accessions (*Indica* Diversity Panel2 and *Indica* Diversity Panel3), a set (*n* = 239) of *Japonica* rice accessions (*Japonica* Diversity Panel), IRRI breeding lines (*n* = 106) and a set of premium rice varieties (*n* = 11) were also selected for validation purposes. *Indica* Diversity Panel2 and *Indica* Diversity Panel3 were grown during the dry season of 2015 and wet season of 2014, respectively, while the *Japonica* Diversity Panel was grown during the dry season of 2015. The IRRI Breeding Lines were grown during the dry season of 2015 and wet season of 2016, while the Premium Varieties were hand-picked from all other sets of accessions.

### 2.2 CEQ indicators

The amylose was determined using the ISO 6647-2-2011 standard iodine colorimetric method using San++ Segmented Flow Analyser (SFA) system (Scalar analytical B.V., AA Breda, Netherlands) (ISO, 2007a, 2007b; Molina et al., 2019). A 100-mg test portion of rice flour was suspended in 1.0 mL 95 % ethanol followed by the addition of 9.0 mL of 1.0 N NaOH. The suspension was heated in a boiling water bath (95 °C) for 10 min to gelatinize. The gel was cooled to room temperature and diluted to 100 mL with deionized (DI) water. The sample was reacted with an aqueous solution of 10 % CH_3_COOH (1.0 N) and 30 % KI-I_2_ (2 %:0.2 %) and the absorbance of the amylose-iodine complex was measured at 620 nm wavelength. It was quantified using a standard calibration curve prepared from reference rice varieties of known ACs (IR65, IR24, IR64, and IR8)

Differential Scanning Calorimetry (DSC) Q100 instrument (TA Instrument, New Castle, DE, USA) was used to capture the GT of each sample (Cuevas et al., 2010). Four milligrams of rice flour was immersed in 8 mg of Millipore water in hermetically sealed aluminum pans. The samples were heated from 25 to 120 °C with an increment of 10 °C per minute. The value of GT was obtained from the temperature of the endothermic peak of the thermogram.

The GC was determined by mixing 100 mg rice flour with 0.2 mL ethyl alcohol containing 0.025 % thymol blue and 2 mL of 0.2 M KOH in a sample tube. The solution was heated in boiling water bath for 8 min then cooled down in an ice-water bath and immediately laid down horizontally on the table for one hour (Molina et al., 2019). GC was measured by the length of the cold paste inside the tube and was compared with the hard (IR48), medium (PSBRC9) and soft (IR42) GC standards.

RVA (Model 4-D, Newport Scientific, Warriewood, Australia) was used to measure the viscosity changes during a heat (50 °C)-hold (95 °C)-cool (50 °C) process as described in the AACC method 61-02 (AACC, 2000). Three grams of rice flour was suspended in 25 g reverse osmosis-purified (RO) water in a canister. Data was collected and processed using ThermoCline for Windows (TCW) version 2.6. A viscosity profile curve was obtained showing the values for pasting temperature (PsT), peak time (PkT), peak viscosity (PV), trough viscosity (TV), and final viscosity (FV). The breakdown (BD), setback (SB), and lift-off (LO) computed by the software (Bao, 2008).

Fifty milligrams of rice flour was gelatinized then debranched at 50 °C for 2 h with 500U/mL of isoamylase (Pseudomonas, Megazyme, Wicklow, Ireland) with consistent agitation. A 40 μL aliquot of debranched solution was analyzed using size exclusion chromatography (SEC) equipped with Ultrahydrogel 250 column (Waters, Alliance 2695, Waters, Millford, USA) to estimate amylose and amylopectin fractions (Ward et al., 2006).

### 2.3 Clustering and modeling of CEQ ideotypes

All the multivariate and statistical analyses were carried out using R software (Version 3.3.2, released 2016). Before choosing an appropriate method of clustering, the clustering tendency of the dataset was assessed (Adolfsson, Ackerman, & Brownstein, 2019). Hartigan’s dip test for pairwise distances was used to check the clustering tendency of the data set. It checks if the pairwise distances of the data are sufficiently different from the uniform distribution. The dataset is clusterable if the p-value of the result is less than 0.05 (Freeman & Dale, 2013; Xu, Bedrick, Hanson, & Restrepo, 2014). Three clustering methods were used to create the CEQ ideotypes based on routine data: Agglomerative nesting using Ward’s method (AGNES), Divisive analysis (DIANA) and k-means clustering. The clusters created were validated via three internal validation measures (silhouette width, Dunn index, and connectivity) and three stability measures (average proportion of non-overlap, average distance, average distance between means, and figure of merit) to conclude the best fitting method (Lange, Roth, Braun, & Buhmann, 2004). The RVA data were used to classify the dataset into a more comprehensive cooking quality ideotypes using the best method assessed. Principal component analysis (PCA) was performed to see if there is distinct separation between clusters and compare how each of the variable used affects each cluster. The created classes were concluded as the cooking quality ideotypes for the selected lines.

To classify each line to a certain ideotype, the RVA parameters were subjected to Random Forest (RF) model. RF model classifier is widely used as classification model for non-linear data due to its accuracy and speed (Dadgar & Brunnett, 2018; Narasimhamurthy & Kumar, 2017). It uses bootstrapping technique to allocate an input (*x_i_*) to a certain class based on majority rule from all groups of tree-based classifiers *h(x_i_, Θ_k_, k = 1,…)*, where *Θ_k_* are independent and identically distributed random vectors (Tatsumi, Yamashiki, Torres, & Taipe, 2015).

Dimension reduction through feature selection was done to avoid overfitting to the model. A correlation filter of 0.75 (r>0.75 and r<−0.75) was used to determine the redundant variables (Yang et al., 2016). Before using the variables resulted from the correlation filter as input to the RF model, their usefulness in the model was checked using the Boruta variable selection method. This method is used exclusively for RF models wherein the variables were randomly permuted to the model via holdout approach of importance measure (Speiser, Miller, Tooze, & Ip, 2019). The data set was split into training and validation set (90/10 ratio) and the RF model optimized to 280 trees (*n_tree_*) with 5 variables or nodes randomly selected at each split (*m_split_*) was used for predicting the classes because it shown the model accuracy. The RF was also used to generate the variable importance for classification into the generated clusters. That is, when a variable gives a higher magnitude of increase in prediction accuracy, it is determined more important (Louppe, Wehenkel, Sutera, & Geurts, 2013). The performance of the resulting classification model was evaluated using the mean decrease in accuracy measure computed from confusion matrix. It was identified through out-of-bag (OOB) subsampling for predicting classification errors wherein the variable importance (*x_j_*) is permuted and the OOB error is adjusted based on difference to reach a minimum value (Hur, Ihm, & Park, 2017). It is computed using the equation

(1) $$VI\left(x_{j}\right)=\frac{1}{n_{tree}}\sum_{t=1}^{n_{tree}}\frac{\sum_{i\in OOB}I\left(y_{i}=f\left(x_{i}\right)\right)-\;\sum_{i\in OOB}I\left(y_{i}=f\left(x_{i}^{j}\right)\right)}{\left|OOB\right|}$$

wherein, *t* is the number of trees from 1 to *n_tree_*, *y_i_=f(x_i_)* is the predicted class before permutation and $y_{i}=f\left(x_{i}^{j}\right)$ is the predicted class after permutation. Furthermore, the reliability of the model was measured using Cohen’s kappa value (κ) for the agreement of predictions (Eq. (2)).

(2) $$\kappa =\;\frac{\left(P\left(a\right)\mathrm{-P}\left(e\right)\right)}{\mathrm{1-P}\left(e\right)}$$

wherein *P(a)* is the percent agreement while *P(e)* is the probability between the observed and predicted values. The κ value represents the agreement between the expected and observed results from the model via random chance (McHugh, 2012). Kappa values less than or equal to zero indicates no agreement, while those in range of 0.01–0.20 has none to slight, 0.21–0.40 has fair, 0.41–0.60 has moderate, 0.61–0.80 has substantial and 0.81–1.00 ha s perfect agreement (McHugh, 2012; Tatsumi et al., 2015). The variable importance of the individual CEQ classes was also obtained by getting the weight contribution of each variable per CEQ class to the over-all mean decrease in accuracy. The model was further cross-validated using *Indica* Diversity Panel2, *Indica* Diversity Panel3, *Japonica* Diversity Panel, IRRI Breeding Lines, and Premium Varieties to check its generalizability.

To develop a comprehensive CEQ models, another RF model was created using the starch structure SEC data. It serves as a second layer model to further classify each ideotype to different sub-classifications. The process of generating results was the same as the first layer of the RF model, although the second layer model used the results from the first model to generate classification. In other words, the first input must be on the first layer before going through the second layer of the classification model.

After creating the two-layered model, the model validity was checked by applying the combined data sets of all the diversity panels to recreate each layer of the RF model. The input variables were again optimized by correlation filter (|spearman rank coefficient| >0.05) and the hyper parameters such as the maximum depth of the forest, maximum number of features to be considered minimum number of trees and sample split were obtained using grid search. The accuracy of the models was then recalculated to check its validity.

### 2.4 Genome-phenome analysis and random forest modeling

We used PLINK for large scale analysis, SnpEff for genetic variant annotation and functional effect prediction and TASSEL for conducting genome wide association studies (GWAS) with filtering criteria of minor allele frequency of 0.05 to identify the effect and top performing SNPs. We conducted RF classification on SNP sets with varying degrees of effect. The primary predictor being the 1st layer cluster, this being a categorical variable, we could not directly associate SNPs so we identified the SNPs associated with each of the 11 traits. With an in intention of identifying the minimum number of SNPs required to get the best predictive accuracy for each of the 11 traits, top 10, top 100 and top 1000 SNP sets were identified based on the P-value cut-offs. Random Forest (RF), a decision tree based algorithm was used to train, test and predict the data. Python based SKLearn machine learning libraries were used to implement RF. The RandomForestClassifier function provided in the SKLearn library was used as a classifier by splitting the sample data set into training (80 %) with test samples (20 %).

### 2.5 Sensory evaluation

A set of samples (*n =* 110) from the 2014 accessions was chosen to undertake sensory evaluation for capturing texture properties. The grains from each sample were cooked as prescribed (Cuevas et al., 2018). Trained set of panelists were recruited to evaluate the texture profile of the samples based on cohesiveness (COH), cohesiveness of mass (COM), hardness (HRD), initial starchy coating (ISC), moisture absorption (MAB), residual loose particles (RLP), roughness (ROF), slickness (SLK), springiness (SPR), stickiness between grains (SBG), stickiness to the lips (STL), toothpack (TPK) and uniformity of bite (UOB). The training phase for the panelist includes difference test, sample and method familiarization and lexicon adjustments based on the panelists’ contexts (Champagne et al., 2010) wherein the rice samples used were commercially available milled rice such as Sinandomeng, Jasmine and Long Grain Rice. The median scores were calculated for each attribute and the profile to describe each ideotype was created through a wheel chart. A lexicon to describe the maximum and minimum values of each attribute was created for easy understanding of these attributes. This is necessary to establish which sensory properties perceived by the consumer describes an ideotype with specific instrumental characteristics. Through this a bridge between the sensory texture parameters known to the consumer, and the instrumental data for aiding high-throughput selections to the breeders, could be established.

The scores were correlated to the routine and RVA properties to see which sensory parameters are affecting, directly or indirectly through Path Coefficient Analysis (Sofiya, Eswaran, & Silambarasan, 2020). Each coefficient which would tell the effect of an independent variable (*i*) to a dependent variable (*j*) were computed using Eq. (3)

(3) *r_i,j_ = P_i,j_* + *Σr_i,k_p_k,j_*

where, *r_i,j_* is the mutual association between the traits, *P_i,j_* is the component of the direct effects of *i* to *j* and the term *Σr_i,k_p_k,j_* is the summation of the components of indirect effects of *i* to *j* via all other independent traits (*k).*

## 3 Results

### 3.1 Rice diversity lines for CEQ characteristics

**Fig. 1:**
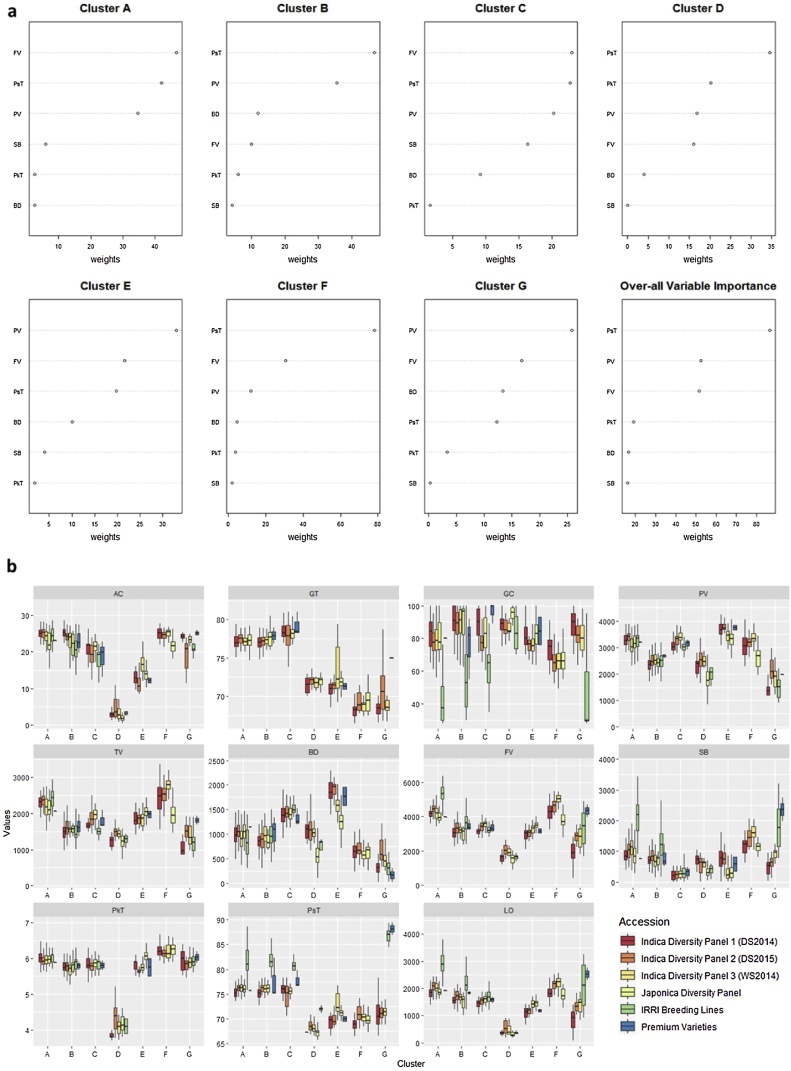
Classification modeling based on the RVA properties using Random Forest. (a) Important variables resulted from modeling based on mean decrease in accuracy and individual decrease in accuracy of each cluster. (b) Phenotypic distribution of selected lines from dry season of 2014 (Indica Diversity Panel 1, *n = 301*), 2015 (Indica Diversity Panel 2, *n = 316*), wet season of 2014 (Indica Diversity Panel 3, *n = 318*), japonica variety (Japonica Diversity Panel, *n = 293*) planted during the dry season of 2015, IRRI Breeding Lines (*n = 106)*, and Premium Varieties (*n = 11*) presented as boxplots comparing the seven cluster created based on selected RVA parameters. Cluster labels are as follows: A, B, C, D, E, F, and G; Variable names are follows: amylose content (AC), gelatinization temperature (GT), gel consistency (GC), peak viscosity (PV), trough viscosity (TV), breakdown viscosity (BD), final viscosity (FV), setback viscosity (SB), peak time (PkT), pasting temperature (PsT) and lift-off viscosity (LO), AM1 (Amylose 1), AM2 (Long-chain Amylopectin), MCAP (Medium-chain Amylopectin), SCAP (Short-chain amylopectin).

The 1741 milled samples comprising three different *Indica* diversity panels, a set of *Japonica* diversity panel (*n* = 239), IRRI breeding lines (*n* = 106) and premium rice varieties (*n* = 11) were subjected to detailed grain quality analysis. The samples represent a huge variation for amylose content ranging from waxy (0.8 %) to high AC (32.60 %), hard to soft GC (28−100 mm) and low (66.4 °C) to high (81.86 °C) GT (Table 1). Using routine grain quality traits only three classes were distinguished using the combinations of AC, GC and GT data (Fig. 1 in Buenafe, Kamanduri, & Sreenivasulu, 2021). Therefore these three parameters routinely used for selecting textural preferences in breeding selection process do not clearly differentiate the CEQ classes within intermediate to high AC group. To be able to fully capture the CEQ of rice reflecting the cooking behavior of rice, the RVA pasting properties were measured. The RVA parameters exhibit wide range of variation for viscosity properties for the entire collection of germplasm (Table 1). Since *Indica* diversity panel1 exhibited similar range of variation as of whole population, we deployed this set to delineate the correlation matrix, derived the seven ideotypes (cluster groups) through AGNES using the RVA properties (Fig. 2 in Buenafe et al., 2021) and developed the CEQ models. The other diversity panels and breeding lines were used to validate the models.

**Table 1:** Phenotypic distribution of all data sets used in the study.

	Routine quality Parameters			Pasting Properties from RVA							
Data Set	AC	GT	GC	PV	TV	BD	FV	SB	PkT	PsT	LO
Indica Diversity Panel 1 (DS2014)	1.3−32.6	66.5−81.7	58.0−100.0	131.8−4248.0	107.6−3363.0	24.2−2291.0	185.4−5353.0	2.0−1666.0	3.7−7.0	65.7−80.6	77.8−2388.0
Indica Diversity Panel 2 (DS2015)	1.6−28.3	66.7−81.8	52.5−100.0	1029.5−4181.3	682.3−3065.8	292.5−2154.3	870.3−5473.5	11.8−2047.8	3.8−6.4	66.9−78.7	188.0−2510.8
Indica Diversity Panel 3 (WS2014)	0.8−27.8	66.7−81.0	46.7−100.0	1223.3−3906.7	980.0−3263.3	243.3−2156.0	1629.0−5439.0	8.0−2027.7	3.7−6.6	66.5−78.9	285.7−2682.5
Japonica Diversity Panel	8.6−26.8	66.8−79.4	43.0−100.0	2415.0−5918.0	1633.0−2824.0	239.0−3656.0	2848.0−4864.0	0.0−2139.0	5.2−6.6	65.7−75.2	1047.0−2177.0
IRRI Breeding Lines	2.6-28.6	N/A	28.0-100.0	919.0-3985.0	775.0-2947.0	32.0-2157.0	1381.0-7060.0	18.0-3833.0	3.9-6.5	71.3-89.3	304.0-4414.0
Premium Varieties	11.5−27.4	70.9−81.0	55.0−100.0	1961.0−3844.0	1368.0−2099.0	25.0−1955.0	3032.0−4701.0	77.0−2740.0	5.5−6.2	69.6−89.4	1143.0−2765.0

Number Distribution Function of Starch Polymers from SEC						
Data Set	AM1 (×10^−12^)	AM2 (×10^−8^)	MCAP (×10^−6^)	SCAP1 (×10^−5^)	SCAP2 (×10^−5^)	SCAP3 (×10^−5^)
Indica Diversity Panel 1 (DS2014)	9.3−6220.0	4.5−28.1	8.7−15.3	4.0−6.8	8.7−18.6	5.7−21.8
Indica Diversity Panel 2 (DS2015)	14.4−10600.0	5.9−51.5	2.6−19.3	1.0−7.8	2.5−16.6	2.2−31.3
IRRI Breeding Lines	24.0−5640.0	13.8−27.2	5.0−235.0	1.9−4.3	4.9−11.1	13.3−31.3
Premium Varieties	1120.0−6720.0	13.6−48.5	11.1−161.0	3.3−5.9	5.3−16.5	2.2−21.5

Abbreviations used: Amylose content (AC), gelatinization temperature (GT), gel consistency (GC), peak viscosity (PV), trough viscosity (TV), breakdown viscosity (BD), final viscosity (FV), setback viscosity (SB), peak time (PkT), pasting temperature (PsT) and lift-off viscosity (LO), AM1 (Amylose 1), AM2 (Long-chain Amylopectin), MCAP (Medium-chain Amylopectin), SCAP1(Short-chain amylopectin, 36 > DP > 21), SCAP2(Short-chain amylopectin, 20 > DP > 13), SCAP3(Short-chain amylopectin, 12 > DP > 6).

### 3.2 Cooking quality model

The pasting properties of rice starch measured using RVA reflects the viscosity (Thin→Viscous) and textural attributes such as hardness (Soft→Firm→Hard). In this study, RF model was implemented to RVA parameters generated from the *Indica* diversity panel1. The cooking quality model showed that FV, BD, PV, SB, and PsT are important variables in differentiating the seven CEQ ideotypes, with an overall accuracy of the model predicted at 96.43 % (Table 1). The RVA models classified selected *Indica* lines from the diversity panel1 fitting to seven ideotype classes as defined by the clustering. The high amylose ideotypes are clearly distinguished based on the weights with different order of RVA parameters, namely group A (FV, PsT, PV), group B (PsT, PV, BD), group F (PsT, FV, PV) and group G (PV, FV, BD) (Fig. 1a). The low or zero amylose ideotype D is characterized by the PsT, PkT, PV variables. The validation of the model from the RVA data generated from *Indica* diversity panel 2 and 3 was found to be very high with accuracy of 81.01 % and 77.67 %, respectively (Table 2). In addition, the cooking model was extended to *Japonica* subspecies with accuracy of 75.43 (Table 2). Results also showed that there were no representative samples predicted from ideotype G in *Japonica* dataset and could not predict ideotype C for the *Indica* diversity panel3 grown in wet season (Fig. 1b). Cohen’s kappa value (κ) for the agreement of predictions (Table 2) was found to be substantially higher (κ 0.61−0.80) and in perfect (κ 0.81–1.00) (McHugh, 2012) agreement within the predicted true value ranges. These results reinforce that models can be applied to any year, season and for varietal predictions in both *Indica* and *Japonica* sub species.

**Table 2:** Validation and accuracy of the CEQ ideotypes from the prediction models.

Models	Over-all Accuracy	Overall Cohen’s kappa value (κ)	Out-of-Bags (OOB) error	Validation Set	Accuracy of Validation Set	Cohen’s kappa value (κ)
First Layer Model (RVA Properties)	96.43 %	0.9522	4.4 %	2015 Dry Season	81.01 %	0.7504
				2015 Wet Season	77.67 %	0.6957
				Japonica	75.43 %	0.6793
Second Layer Model-Ideotype A	100 %	0.9998	7.69 %	2015 Dry Season	68.83 %	0.5234
Second Layer Model-Ideotype B	100 %	0.9997	1.33 %	2015 Dry Season	77.88 %	0.7012
Second Layer Model-Ideotype F	100 %	0.9996	4.35 %	2015 Dry Season	57.89 %	0.4832

**Fig. 2:**
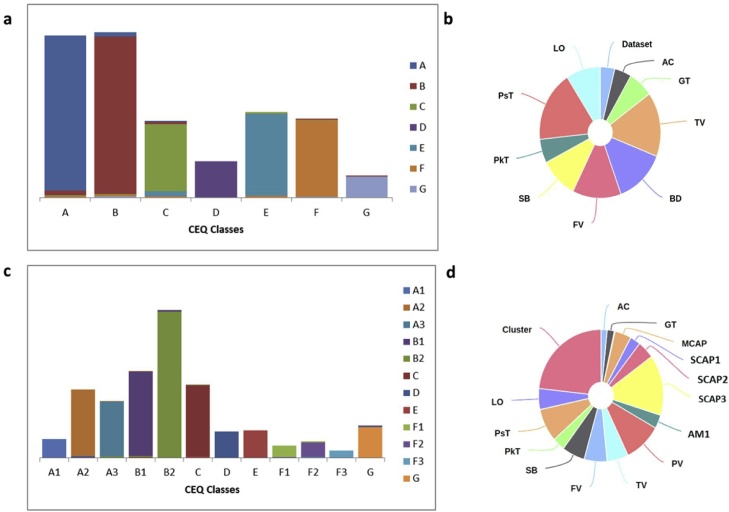
Results of Validating the Model using the combined data sets. (a) Confusion bar plots for the first layer of the Random Forest Model. (b) Distribution of variable importance of the first layer of the model. (c) Confusion bar plots for the second layer of the Random Forest Model. (d) Distribution of variable importance of the second layer of the model. Variable names are follows: amylose content (AC), gelatinization temperature (GT), gel consistency (GC), peak viscosity (PV), trough viscosity (TV), breakdown viscosity (BD), final viscosity (FV), setback viscosity (SB), peak time (PkT), pasting temperature (PsT) and lift-off viscosity (LO), AM1 (Amylose 1), AM2 (Long-chain Amylopectin), MCAP (Medium-chain Amylopectin), SCAP1(Short-chain amylopectin, 36 > DP > 21), SCAP2(Short-chain amylopectin, 20 > DP > 13), SCAP3(Short-chain amylopectin, 12 > DP > 6).

In order to validate the model outputs, we have combined all six datasets that comprised 1741 samples with a split of 1390 training and 348 test samples and predicted the seven CEQ ideotypes with an accuracy of 0.91 using random forest classifiers. The derived confusion matrix neatly classified 7 CEQ groups with limited mismatches (Fig. 2a). The model shows that while PsT, TV, FV, BD, SB, LO were identified as important features in predicting 7 CEQ groups, the PkT, GT and AC made minor contribution (Fig. 2b).

**Fig. 3:**
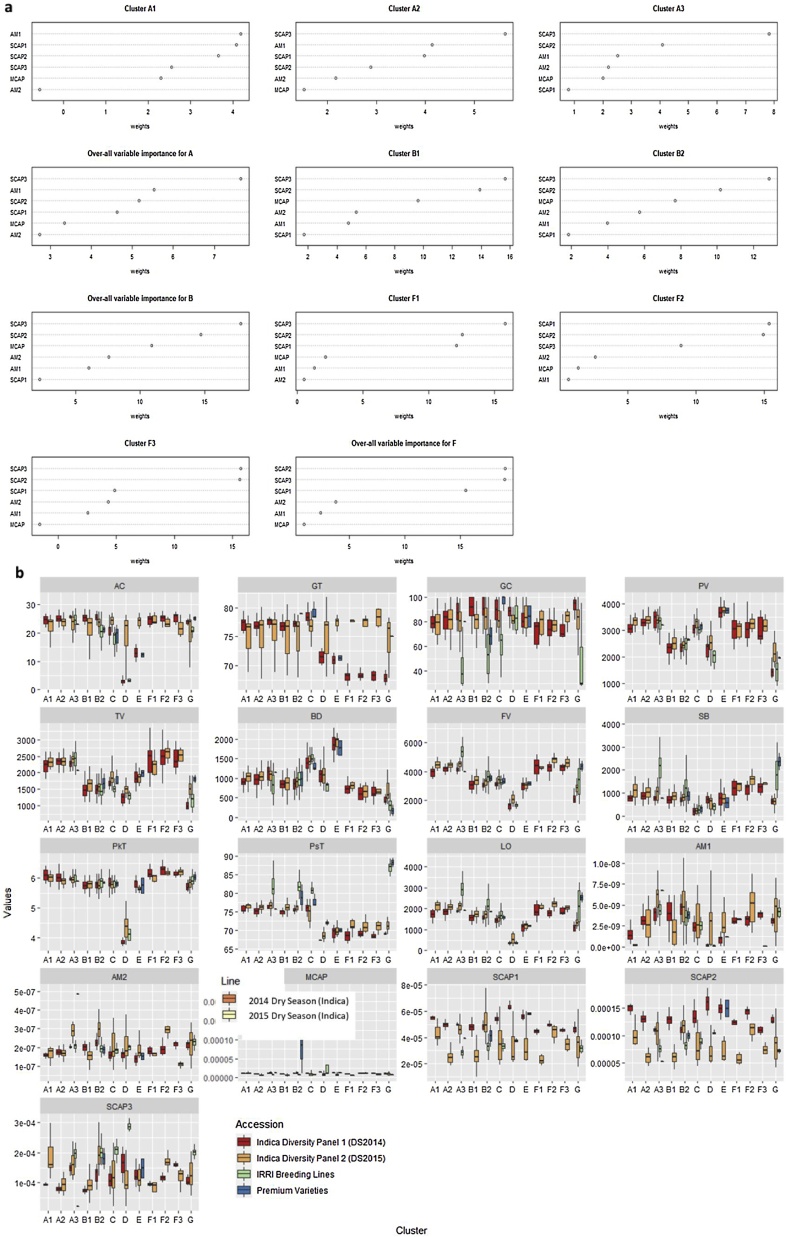
Classification modeling based on the SEC properties using Random Forest. (a) Important variables resulted from modeling based on mean decrease in accuracy and individual decrease in accuracy of each cluster. (b) Phenotypic distribution of selected lines from dry season of 2014 (Indica Diversity Panel 1, *n = 301*), 2015 (Indica Diversity Panel 2, *n = 316*), IRRI Breeding Lines (*n = 106)*, and Premium Varieties (*n = 11*) presented as boxplots comparing the seven cluster created based on selected RVA parameters. Cluster labels are as follows: A, B, C, D, E, F, and G; Variable names are follows: amylose content (AC), gelatinization temperature (GT), gel consistency (GC), peak viscosity (PV), trough viscosity (TV), breakdown viscosity (BD), final viscosity (FV), setback viscosity (SB), peak time (PkT), pasting temperature (PsT) and lift-off viscosity (LO), AM1 (Amylose 1), AM2 (Long-chain Amylopectin), MCAP (Medium-chain Amylopectin), SCAP1(Short-chain amylopectin, 36 > DP > 21), SCAP2(Short-chain amylopectin, 20 > DP > 13), SCAP3(Short-chain amylopectin, 12 > DP > 6).

Unravelling the exact composition of amylose and amylopectin variation (starch structure properties) is critical to capture the linkages between CEQ and textural attributes. The molecular size of amylopectin structures was found to have high correlations with all the RVA properties (Kowittaya & Lumdubwong, 2014). Hence the number distribution function (P(M)) of each starch polymer structure was used to derive the second degree of modeling to predict sub-types of CEQ ideotypes by accounting variation in amylose 1 (AM1, degree of polymers DP > 1000), long-chain amylopectin (AM2, DP 121–100), medium-chain amylopectin (MCAP, DP 37–120), and three polymers of short chain amylopectin (SCAP1, SCAP2, and SCAP3 found at DP 21–36, DP 13–20, and DP 6–12, respectively). The relative importance of each variable was identified per sub-cluster. The P(M) values for SCAP3 and SCAP2 are among the top priorities for the accuracy of the models for A, B and F (Fig. 3a). Ideotype A were further subdivided into three (A1, A2, and A3) while B and F were subdivided into two (B1 and B2) and three (F1, F2, and F3) clusters, respectively. This comprehensive cooking quality prediction resulted to the identification of a total of twelve ideotypes (Fig. 3b). The combined models developed from RVA derived parameters and starch structural properties from 798 samples of *indica* germplasm predicted 12 ideotypes wherein primarily cluster information was included with auto search hyperparameter grid. We recorded an accuracy of 93.5 % with a split of 638 training and 160 test samples. In attempt to remove bias created by primary cluster, we remodeled without primary cluster info and with a slightly reduced accuracy in predicting sub clusters at approximately 85 %. The models projected the importance of SCAP3 (degree of polymers-DP 6–12), PsT, TV, FV, BD, SB and LO as important salient features in predicting the 12 ideotypes (Figs. 2b, 3 in Buenafe et al., 2021). When we considered alone starch structure data to sub-classify the ideotypes A2, A3, B1, B2 and F1, SCAP3 was identified as the most important variable for classification; while ideotype A1 and F2 was characterized with AM1 and SCAP1 starch fraction as the most important variables (Fig. 3a). The applicability of the model was validated by data generated from independent *Indica* core collection panel grown in dry season of another year (Table 2) and the κ for the agreement of predictions was found to have substantial agreement within the predicted and true values (Table 2), which shows that the model can be applied to the independent years to predict cooking quality.

### 3.3 Sensory characteristics of CEQ ideotypes

**Fig. 4:**
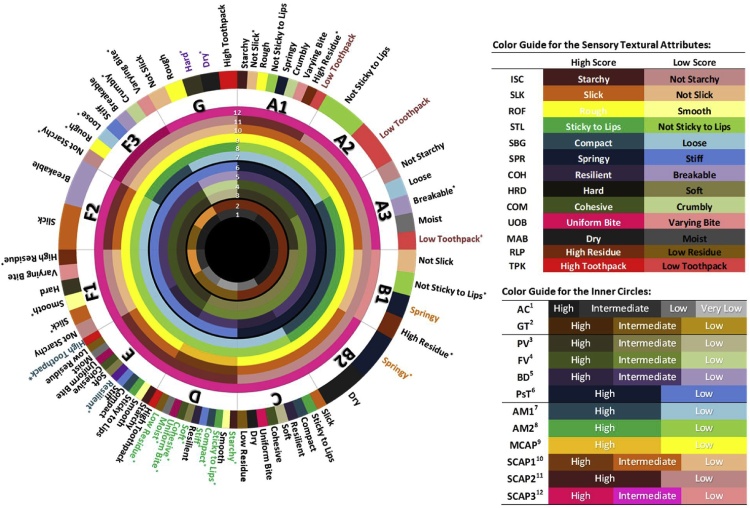
Rice texture wheel chart for each clusters with their corresponding sensory descriptions. The description in the outer circle highlighted in colors is the sensory description for each ideotype and the wheel chart also features some of the routine quality, RVA, and starch structure parameters that are deemed important both in modeling and classification. The sensory characteristics in the wheel chart marked with an asterisk (*) was the ideotype which received either the minimum or the maximum score in that particular attribute. For example A1 has the lowest score for slickness, while F1 got the highest score for the same attribute. Variable names are follows: amylose content (AC), gelatinization temperature (GT), gel consistency (GC), peak viscosity (PV), trough viscosity (TV), breakdown viscosity (BD), final viscosity (FV), setback viscosity (SB), peak time (PkT), pasting temperature (PsT) and lift-off viscosity (LO), AM1 (Amylose 1), AM2 (Long-chain Amylopectin), MCAP (Medium-chain Amylopectin), SCAP1(Short-chain amylopectin, 36 > DP > 21), SCAP2(Short-chain amylopectin, 20 > DP > 13), SCAP3(Short-chain amylopectin, 12 > DP > 6), initial starchy coating (ISC), slickness (SLK), roughness (ROF), stickiness to lips (STL), stickiness between grains (SBG), springiness (SPR), cohesiveness (COH), hardness (HRD), cohesiveness of mass (COM), uniformity of bite (UOB), moisture absorption (MAB), residual loose particles (RLP), and toothpack (TPK) were generated.

Measuring the textural parameters through trained sensory panel is tedious, low throughput but often provides gold standard data. More than one hundred lines identified through bi-layered modeling representing the twelve ideotypes of cooking quality were subjected to the tasting panelists to describe 13 textural properties of sensory profiles (Fig. 4, Table 1 in Buenafe et al., 2021). The path-coefficient analysis emphasized the importance of RVA parameters and starch properties with sensory textural attributes (Fig. 4 in Buenafe et al., 2021).

The sensory profile of 12 defined ideotypes shown in a sensory wheel chart created by getting the top three highest and lowest scores of each of the sensory textural attributes represented in each ideotype (Fig. 4). The relationship of the sensory parameters observed in the wheel chart depict that ideotypes having very low to low AC (C,D, and E) tends to be sticky to lips, compact, soft, cohesive, and low residual loose particles. Generally, ideotypes having very low amylose content (D and E) have higher stickiness to lips and between the grains (STL and SBG). The panel detected that these two classes have more ISC, higher STL and SBG, lower HRD, higher COM, UOB and lower RLP. The only difference between the two is that ideotype D tends to have higher scores for ISC, STL, SBG, COM, and UOB than E. This is expected since ideotype D contains lower amylose content than E. The ideotype E has the highest level of COH and TPK.

Although the lines represented in A, B, F and G ideotypes were found to be high AC in nature, they are linked to unique sensory properties (Fig. 4). Lines belonging to ideotype A (A1, A2, and A3) have low toothpack and these sub-clusters could be further distinguished with unique textural attributes such as A1 possessing non-slick, high RLP and A3 ideotype with breakable cohesive property. Though ideotypes B1 and B2 have springy texture, ideotype B2 has the highest level of springiness (SPR). While ideotype A1, B1 and F1 has the highest levels of RLP; ideotype F1 were distinguished with the highest levels of SLK and ideotype F3 with the highest level of ROF. The ideotypes having low GT (F1 and F3) are not starchy and has varying bite. Ideotype F3 with high P(M) value for SCAP3 are stiff (low springiness) while ideotype B1 with low P(M) value for SCAP3 are springy in nature. Ideotypes with high MCAP P(M) values tends to have high values for ISC, STL, SBG, COM, and UOB and low values for SPR, HRD, MAB, and RLP. Ideotype G was the most unique ideotype among all the clusters found to have the highest level of HRD and MAB, characterized as being hard and dry. Ideotype G with low BD is hard and ideotype C and E with high BD are soft textured (Fig. 4).

### 3.4 Genotype data modeling to predict the CEQ ideotypes

We have conducted genome wide association studies (GWAS) to link the genotype data with phenotype data of routine grain quality traits (AC, GC, GT) and RVA parameters (PV, TV, BD, FV, SB, PkT, PsT and LO) using TASSEL software package. From 1.8 million single nucleotide polymorphisms (SNPs) dataset, we observed 8,437,253 associations (767,024 unique SNPs) with the AC, GC, GT, PV, TV, BD, FV, SB, PkT, PsT and LO phenotypes of interest and we filtered the top 10, 100, 1000 SNPs (for each phenotype) based on the p-value threshold from TASSEL. RF modeling was performed on each of these top 10, 100 and 1000 SNP set (9538 unique SNPs associated with the 11 traits) resulting in an accuracy prediction of 0.51, 0.55 and 0.68, respectively. Among it, the first exon/intron boundary SNP a highly significant T→G splice variant at 1 765 761 bp distinguished waxy genotypes from non-waxy (Anacleto et al., 2019).

We independently conducted RF modeling on the full 1.8 million SNPs that provided us with a list of most influential features for a target predictor. Upon remodeling with RF by considering only the top 1000 SNPs (important features) from the initial 1.8 million SNP model, for the 1st layer cluster as target variables, 7 ideotypes (A to G) were neatly classified with a good accuracy at 0.81.

In order to remove scope for bias, we randomly selected samples to show equal representations of clusters from A to G. With cluster ‘G’ having the least number of samples associated (64), we took that as the baseline and created a dataset of 452 samples (with equal number of samples across clusters ‘A’ to ‘G’). In order to check if effective genotypes could be identified that could increase predictive accuracy using KNN models. Parallel, we took top 1k SNPs that were most influential when random forest algorithms were run for genome-phenome analysis and applied to build KNN models which provided best predictive accuracy at 0.89 %.

Alternative modeling was also performed for top 10 and top 100 SNPs, but they did not yield good results as accuracy levels were below 0.5. The functional annotation of these top 1000 SNPs identified genes belongs to major functional categories of protein degradation, transcription factors and signaling receptor kinase. One third of these SNPs cover starch metabolism, cell wall metabolism, lipid metabolism, secondary metabolism, cytochrome P450 and stress related genes.

### 3.5 Predicting the CEQ of IRRI’s breeding material

**Fig. 5:**
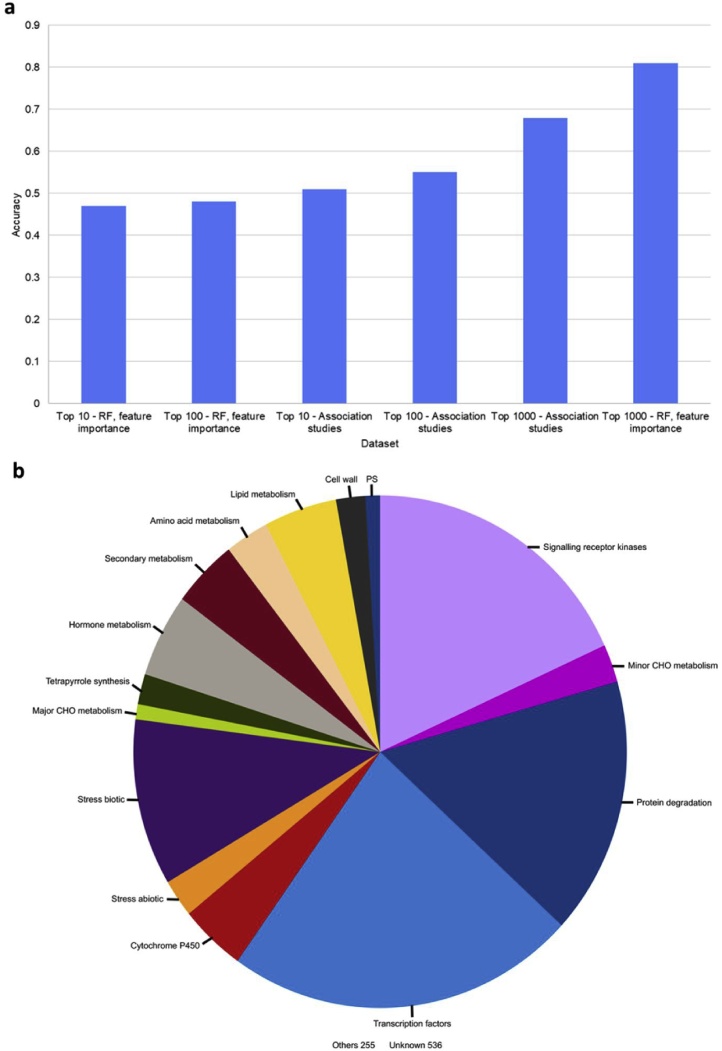
Results of GWAS linking the genotype and phenotype of the *Indica* Diversity Panels. (a) Accuracy plot of GWAS and Random Forest (RF) models using the threshold of considering the top 10, 100 and 1000 SNPs. (b) Functional categories of top 1000 SNPs identified using RF model to classify the 7 ideotypes.

**Fig. 6:**
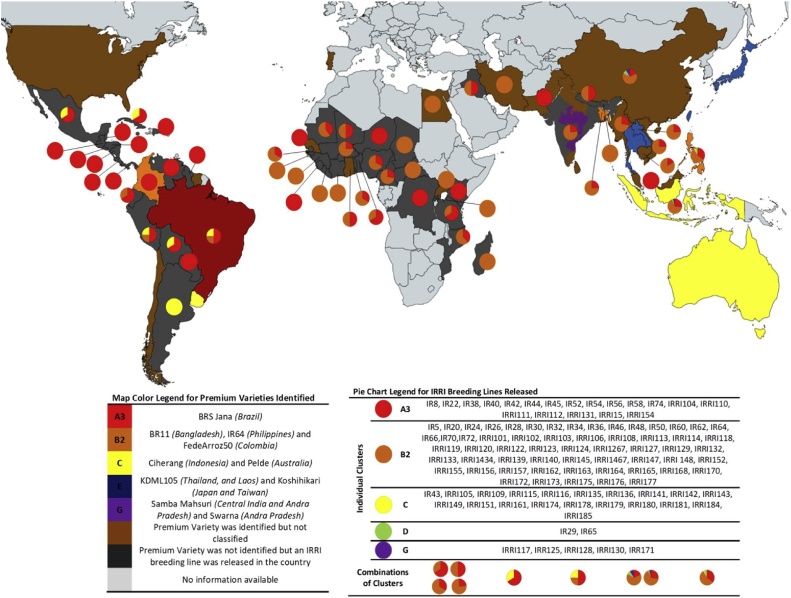
Geographical distribution of released IRRI Breeding Lines and Premium Varieties per country. Premium varieties per country were identified by Calingacion et al. (2014) according to consumer preferences. Countries without a reflected pie chart means that there was no recorded IRRI Breeding Line released on that country. The map color legend represents the countries that have an identified premium variety classified according to the CEQ classes from the models. The pie charts which show the percentage distribution of IRRI breeding lines matching to distinct ideotypes released in a specific target country is depicted along with its benchmark varieties. Each color in the pie chart represents the CEQ class of the IRRI breeding lines released in that country.

Applying the models to IRRI’s breeding material has predicted only five ideotypes (A3, B2, C, D and G) out of twelve (Fig. 5). Some of the identified premium varieties classified as ideotypes A3 (BRS Jana), B2 (IR64, BR11), C (Ciherang, INIA Tacuari, Pelde), E (Koshihikari and KDML105), or G (Sambha Mahsuri, Swarna). Most breeding lines released in Asia and Africa was under class B2. Among the best fit, in the Philippines IR64 is classified under ideotype B2 fitting to the target preference of B2 ideotype with springy texture. Likewise, Brazil’s BRS Jana is under ideotype A3 and most of the released IRRI breeding lines in their country are classified under ideotype A3 as well. Interestingly, this exercise also identified several gaps in the breeding targets. Central India’s premium varieties, Samba Mahsuri and Swarna, are classified under ideotype G (generally dry and hard) but the released breeding lines in the country’s target zone were classified as either ideotype A3 or ideotype B2. Indonesia’s Ciherang is classified under ideotype C but the breeding line released in their country were ideotypes A3, B2, D, and G. Colombia’s Fedearroz50 is classified as ideotype B2 but the ones released in their country was under ideotype A3. Laos prefers KDML105 which is under ideotype E, which exhibits high toothpack and cohesiveness, but released varieties in their country were classified under A3 and B2 (Fig. 6).

## 4 Discussion

Targeting amylose as selecting criteria in breeding material varieties lead to the development of waxy amylose with sticky rice texture in countries like Lao PDR, low AC with soft texture preferred in Japan, Taiwan, Cambodia, Thailand, Australia, northern china and southern Vietnam (Anacleto et al., 2015). Rice varieties with intermediate to high AC used widely to breed *Indica* germpasm in South Asian countries like Myanmar, Sri Lanka, India, Pakistan and Indonesia differ in its texture (Calingacion et al., 2014), which cannot be captured alone using amylose. Scanning large germplasm of *Indica* lines from IRRI’s breeding program suggest that high amylose lines are also in the vicinity of soft GC suggesting that some of the high-amylose varieties remain soft upon cooling (Anacleto et al., 2015), while others are hard and retrograded. So far we lacked the effective phenotyping techniques to capture metrics associated with pasting properties during cooking processing through RVA and unraveling starch properties through SEC (Bao, 2008; Butardo et al., 2017; Hsu et al., 2014) to be linked with textural properties. RVA is documented to readily differentiate varieties that are of the same amylose class (Wang, Yin, Shen, Xu, & Liu, 2010). The information obtained by RVA have yet to become criteria for releasing new varieties and in evaluating rice traded internationally to capture CEQ in the breeding pool. To address these limitations, we developed holistic tools of modeling to link initial cooking quality indicators (AC, GC and GT) with cooking processing behavior (RVA profiling) and starch quality assessment parameters to capture overall grain quality preferences reflecting CEQ classes and textural preferences within the breeding germplasm.

In the past, several attempts made to create classification models for the water uptake and gelatinization during cooking (Briffaz, Mestres, Matencio, Pons, & Dornier, 2013) but no systematic attempt made to predict the CEQ ideotypes relating to sensory properties with a larger data set covering wide spectrum of variation. Prior models deployed to assess grain quality by comparing support vector machine (SVM), K-nearest neighbors models (Lu & Zhu, 2014), multinomial logistic regression (Cuevas et al., 2018), partial least square discriminant analysis (Cameron & Wang, 2005). However RF models are far more superior to all of the models when it comes to accuracy and sensitivity to the input variables (Statnikov, Wang, & Aliferis, 2008).

The first layer only requires the RVA parameters to classify a rice sample and the model can be broadly classified at this stage. The results might be less comprehensive but it shows decent distinction between the 7 ideotypes, compared to only 3 groups identified with routine grain quality traits. This makes RVA a one-step solution in providing classification for the breeders. The modeling results have shown that high amylose groups (ideotypes A, B, F and G) are neatly classified based on the most important features PV, FV and PsT mostly due to differential resistance potential against swelling of starch granules while being heated which can be attributed to the amylopectin composition (Brites, Santos, Bagulho, & Beirão-da-Costa, 2008; Cornejo-Ramírez et al., 2018; Shafie, Cheng, Lee, & Yiu, 2016).

Previous studies have been conducted to elucidate the genetic bases of the different attributes that predict rice’s CEQ properties. Amylose content and viscosity properties have been associated with the *Waxy* gene, which codes for the Granule-Bound Starch Synthase (GBSS) 1 enzyme (Anacleto et al., 2019). GT has been associated with SNPs Starch Synthase (SS) IIa gene (Parween et al., 2020). The snp_06_1765761 with a T to G change at the 5’ splice site of intron 1 of the first splice variant of *GBSS I* (LOC_Os06g04200.1) known to distinguish waxy (no amylose content) rice from the intermediate to high ones (Anacleto et al., 2019; Yamanaka, Nakamura, Watanabe, & Sato, 2004). It however cannot distinguish waxy from low, and intermediate from high amylose content rice. As explained by the models defined based on the phenotyping data we need to go beyond the amylose by considering the entirety of data to predict the overall CEQ ideotypes. Finding diagnostics molecular markers as selection tools to predict CEQ can fast track selections in the breeding programs. Hence it is important to develop more in-depth knowledge about how different genes affect the CEQ properties of the grain. To do so, we have elucidated the genetic bases of the different attributes that predict rice’s CEQ properties. In this study we used GWAS approach to identify genetic variants for all 11 traits of CEQ resulted in identifying top 1000, 100 and 10 SNP sets. RF modeling using these GWAS derived genetic variants did not yield highly heritable classification suggesting that genetic variants identified through single locus association did not capture the overall heritability of CEQ ideotypes. This limitation was overcome by implementing RF models to test the large set of 1.8 million SNP sets to identify top 1000 SNP variants which explain high interaction effects and capture the high dimensionality of genomic data with a higher prediction accuracy of 0.81. Interestingly these target genes covers not only starch biosynthesis pathway, but covers pathways of cell well metabolism, lipid, amino acid, secondary metabolism, protein degradation and important regulators.

The two-layered nature of the RF models defined based on phenotyping data neatly classifies individual variety CEQ property to 12 ideotypes with higher accuracy as these models are valid across the germplasm of *Indica* and *Japonica* subspecies and as well reproduced across years and seasons with higher accuracy when RVA (PV, FV and PsT as primary factors) and starch properties (SCAP3, SCAP2) are considered jointly. SEC experiments targeted to estimate not only the amylose but also different degree of polymers of amylopectin which contributes to differential texture emphasized the importance of SCAP3. These parameters are proven fast and efficient methods for rice characterization (Pang et al., 2016; Zeng et al., 2017) and were already established to be significantly correlated with sensory qualities of rice (Calingacion et al., 2014; Chandra et al., 2012; Custodio et al., 2019). Results of this study have shown that the RVA properties and starch structure properties can be utilized to distinguish 12 CEQ ideotypes with different sensory textural profiles. These models can be used as a detailed selection tool for screening of a variety that can be included as selection criteria in the breeding programs to cater the needs of both farmers and consumers. By applying the models to IRRI breeding lines, we can now gauge the current stand of these lines in capturing the consumer preferences. A study by Calingacion et al. (2014) identified premium varieties from selected countries. It was found out that Japan, Taiwan, Laos, and Thailand preferred rice that belongs to ideotype E which is generally sticky and soft rice (Fig. 4). However, taking for example Laos, we could see that the IRRI breeding lines release to their country falls under ideotypes A3 and B2 which is a mismatch on what they prefer. Same thing was observed in central parts of India, wherein the most preferred type of rice belongs to class G which is generally hard and dry but the lines released on the target zone were found to be classified as A3 and B2. These mismatches can be addressed in future breeding programs by applying the derived models to capture the CEQ and textural preferences and disseminate the rightly chosen varieties to the target countries by matching the preference of consumers in terms of texture. The RF models developed based on phenotype data and high-density genotyping data will be useful breeding tools to improve CEQ and textural preferences in rice.

## CRediT authorship contribution statement

**Reuben James Q. Buenafe:** Conceptualization, Data curation, Formal analysis, Visualization, Methodology, Writing - original draft. **Vasudev Kumanduri:** Visualization, Validation. **Nese Sreenivasulu:** Conceptualization, Supervision, Funding acquisition, Writing - original draft, Writing - review & editing.

## Declaration of Competing Interest

The authors report no declarations of interest
